# Preparation of CO_2_-Adsorbing Fire-Extinguishing Gel and Study on Inhibition of Coal Spontaneous Combustion

**DOI:** 10.3390/gels12010068

**Published:** 2026-01-12

**Authors:** Jianguo Wang, Zhenzhen Zhang, Conghui Li

**Affiliations:** College of Safety Science and Engineering, Xi’an University of Science and Technology, Xi’an 710054, China; 15529066075@163.com (Z.Z.);

**Keywords:** coal mine fire prevention and control, carbon dioxide adsorption gel, oxygen barrier, thermal stability, flame retardant properties

## Abstract

Spontaneous coal combustion accounts for more than 90% of mine fires, and at the same time, the ‘dual carbon’ strategy requires fire prevention and extinguishing materials to have both low-carbon and environmentally friendly functions. To meet on-site application needs, a composite gel with fast injection, flame retardant, and CO_2_ adsorption functions was developed. PVA-PEI-PAC materials were selected as the gel raw materials, and an orthogonal test with three factors and three levels was used to optimize the gelation time parameters to identify the optimal formulation. The microstructure of the gel, CO_2_ adsorption performance, as well as its inhibition rate of CO, a marker gas of coal spontaneous combustion, and its effect on activation energy were systematically characterized through SEM, isothermal/temperature-programmed/cyclic adsorption experiments, and temperature-programmed gas chromatography. The results show that the optimal gel formulation is 14% PVA, 7% PEI, and 5.5% PAC. The gel microstructure is continuous, dense, and rich in pores, with a CO_2_ adsorption capacity at 30 °C and atmospheric pressure of 0.86 cm^3^/g, maintaining over 76% efficiency after five cycles. Compared with raw coal, a 10% gel addition reduces CO release at 170 °C by 25.97%, and the temperature-programmed experiment shows an average CO inhibition rate of 25% throughout, with apparent activation energy increased by 14.96%. The gel prepared exhibited controllable gelation time, can deeply encapsulate coal, and can efficiently adsorb CO_2_, significantly raising the coal–oxygen reaction energy barrier, providing an integrated technical solution for mine fire prevention and extinguishing with both safety and carbon reduction functions.

## 1. Introduction

During the coal mining process, the risk of mine fires is always present, with over 90% originating from coal spontaneous combustion [1]. With the advancement of mining technology, mining depth and intensity continue to increase, and the range of goaf areas keeps expanding, resulting in aggravated underground air leakage problems. This has led to a significant rise in the frequency of mine fires [2,3,4]. Therefore, continuous attention must be paid to the prevention and control of coal spontaneous combustion by implementing effective fire prevention and extinguishing measures to reduce accident rates and ensure both personnel safety and the sustainable use of coal resources. Meanwhile, in 2020, China set a clear “dual carbon” emission reduction target: to achieve peak CO_2_ emissions before 2030 and carbon neutrality before 2060 [5,6,7]. However, according to the International Energy Agency’s “CO_2_ Emissions in 2023” report, global energy-related CO_2_ emissions in 2023 increased by 1.1% year-on-year (about 410 million tons), with a steady upward trend in CO_2_ emissions from energy combustion and industrial processes over the past decade [8,9,10]. This means that the prevention and control of coal spontaneous combustion must also consider environmental requirements; while suppressing fires, low-carbon measures should be taken to reduce environmental damage caused by coal spontaneous combustion, contributing to the realization of the “dual carbon” goals and creating a better living environment for humanity [11,12,13]. Therefore, the development of effective CO_2_ adsorption-based fire prevention and extinguishing materials is of great significance for the sustainable development of coal mining enterprises.

In recent years, research on CO_2_-adsorptive gel materials has been continuously advancing. Researchers have obtained gel materials with excellent adsorption performance by modifying raw material formulations and introducing new crosslinking agents; improved the CO_2_ adsorption capacity of gels through surface modification and pore structure regulation; and provided a theoretical basis for the practical application of gel adsorbents by investigating the effects of factors such as temperature and pressure on adsorption performance. Zhou et al. [14] compounded the metal–organic framework material Cu-BTC with SiO_2_ aerogel, endowing the composite with a hierarchical micro/mesoporous structure that enhances the physical adsorption of CO_2_ by strengthening intermolecular forces (van der Waals forces); they also used TEPA for impregnation modification to improve the chemical adsorption of CO_2_. Kim, J. Y et al. [15] successfully prepared a sodium polyacrylate-based sodium carbonate hydrogel, which can effectively absorb CO_2_ without adding extra water, and absorbs faster at a low temperature of 10 °C than at room temperature, providing an efficient CO_2_ adsorption solution for the food packaging field. Wang, J et al. [16] from a theoretical computational perspective, the intermolecular interactions between four biodegradable polymers (PEG, PVP, chitosan, and pHEMA) and CO_2_ were studied. Density functional theory calculations revealed that chitosan and polyvinylpyrrolidone models have the strongest adsorption affinity for CO_2_, and the interaction mechanism was elucidated at the molecular orbital level, providing a theoretical basis for designing and synthesizing high-performance, biodegradable CO_2_-adsorbing gel materials. Qian et al. [17] regulated the pore structure and surface charge properties of traditional electrostatic interaction-based self-assembled gels by adjusting parameters such as pH and ionic strength, resulting in a new type of self-assembled gel with high selectivity for N_2_/CO_2_ separation. Valdebenito Fabiola et al. modified nanocellulose aerogels through a silanization reaction using N-[3-(trimethoxysilyl) propyl]ethylenediamine (DAMO), introducing varying amounts of amino groups, and focused on examining the performance decay of these materials over multiple adsorption–desorption cycles. The results showed that CO_2_ adsorption capacity is directly related to the amino group loading on the material. Under conditions of 4 bar pressure and 25 °C, samples with amino loadings of 4.62, 9.24, and 13.87 mmol DAMO exhibited CO_2_ adsorption capacities of 3.17, 5.98, and 7.86 mmol/g, respectively. In summary, existing research on CO_2_-adsorptive gel materials mainly focuses on single adsorption functionality, and multifunctional materials integrating both CO_2_ adsorption and coal fire prevention/extinction capabilities have not yet been developed. Additionally, systematic studies on the synergistic optimization of “gelation controllability-fire suppression stability-CO_2_ adsorption performance” are lacking, making it difficult to meet the integrated demand for “disaster prevention and gas treatment” in coal mine and similar scenarios. As a major greenhouse gas, CO_2_ capture technologies have made certain progress, but their high cost and complex operational processes hinder the application of traditional CO_2_ capture technologies in mine environments [18]. Furthermore, the complex mine conditions (e.g., high temperature, high humidity, and high dust) limit the adaptability and practical effectiveness of related technologies.

Therefore, A multifunctional gel material with both fire prevention and fire extinguishing properties, as well as CO_2_ adsorption capacity, was developed. The gelation time of this gel can be controlled, allowing it to wrap coal for extended periods to achieve fire prevention and extinguishing effects. Firstly, an orthogonal experiment was designed with a three-factor and three-level scheme; subsequently, the optimal gel formulation was determined via significance analysis; finally, the gel’s micromorphology, CO_2_ adsorption capacity, and coal spontaneous combustion inhibition characteristics were analyzed in detail.

## 2. Results and Discussion

### 2.1. Analysis of the Effect of Component Content on Gel Performance

(1) Orthogonal Experimental Design

Using PVA as content A, PEI as content B, and PAC as content C, an orthogonal experimental design was created with these three factors as variables. After preliminary screening, content A was set at three levels: 10%, 12%, and 14%; content B was set at three levels: 7%, 8%, and 9%; content C was set at three levels: 4.5%, 5%, and 5.5%. An orthogonal experimental design for the three factors and three levels mentioned above was established, with the factor levels shown in Table 1.

According to the ratios in Table 1, nine sets of orthogonal experimental formulas were generated using SPSS software version 2023, as shown in Table 2.

The gelation time was measured using the drip timing method. A prepared gel solution was dripped through a funnel, and the time for the dripping to complete was recorded. The steps were repeated until the gel dripping time exceeded 50% of the previous time, at which point this time was considered the gelation time. Each gel sample was tested three times, and the average value was taken to reduce error.

(2) Result Analysis

According to the orthogonal experimental design, 9 groups of gel tests were prepared and the gelation time data were obtained. The results of the orthogonal experiment are shown in Table 3. The test results were analyzed using multi-factor analysis of variance to study the effects of various factors at different levels on the experimental results. In addition, multi-factor analysis can distinguish between the primary and secondary factors affecting the results, making it easier to study the significance of each factor. The variance analysis results were calculated using SPSS software version 2023.

**Figure 1:**
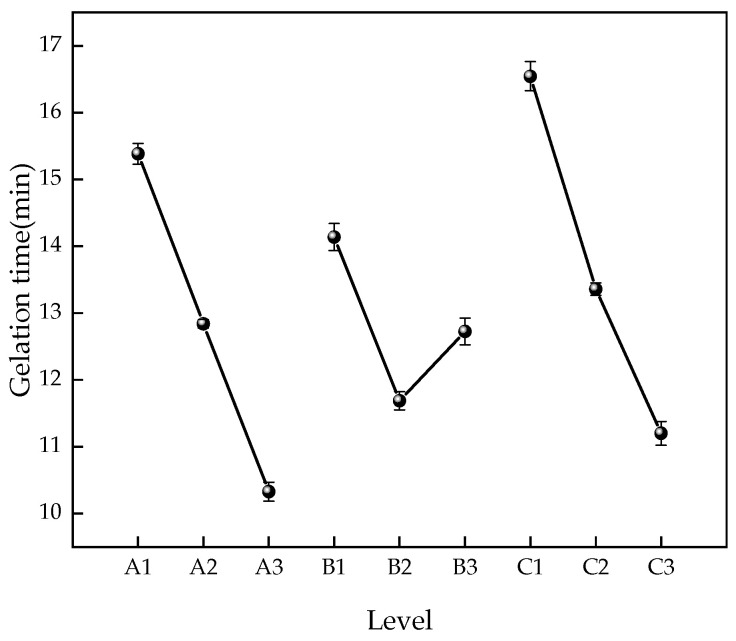
Effect of each factor level on gelation time.

Based on the results of the orthogonal experiment, a statistical analysis was conducted on the effects of different factors and levels on gelation time. The influence of each factor level on gelation time is shown in Figure 1. The results of the significance analysis of each factor are shown in Table 4.

Combined with the chart, it can be seen that the R^2^ value is 0.987, and the calculation results are consistent with reality. PVA showed significance (F = 23.258, *p* = 0.041 < 0.05), indicating a main effect exists, and PVA has a differential effect on gelation time. PEI did not show significance (F = 4.290, *p* = 0.189 > 0.05), indicating that PEI does not have a differential effect on gelation time. PAC showed significance (F = 49.000, *p* = 0.020 < 0.05), indicating a main effect exists, and PAC has a differential effect on gelation time. The influence of the three factors on gelation time is PAC > PVA > PEI. The effect of PEI content on gelation time is relatively small; gelation time is mainly affected by PVA and PAC. Analyzing the effect on gel gelation time allows for the formulation of gels with different gelation times according to the needs of different mines, improving the applicability of gel-based fire prevention materials.

In order to be effectively applied to fire prevention and control in coal mines, the gelation time of the gel should neither be too fast nor too slow. If the gelation time is too fast, the gel cannot penetrate deep into the cracks of the coal seam; if it is too slow, the gel may flow out of the cracks and fail to stay on the coal surface. The gelation time should be controlled within 5–10 min [19]. Four gel formulation ratios were ultimately selected, namely 10% A + 8% B + 5.5% C, 12% A + 9% B + 5.5% C, 14% A + 7% B + 5.5% C, and 14% A + 8% B + 5% C. Subsequent work will involve basic performance testing of gels and optimization of formulations based on these four ratios.

### 2.2. Gel Thermal Stability Analysis

#### 2.2.1. Analysis of Thermal Stability Test Results Under Constant Temperature Conditions

**Figure 2:**
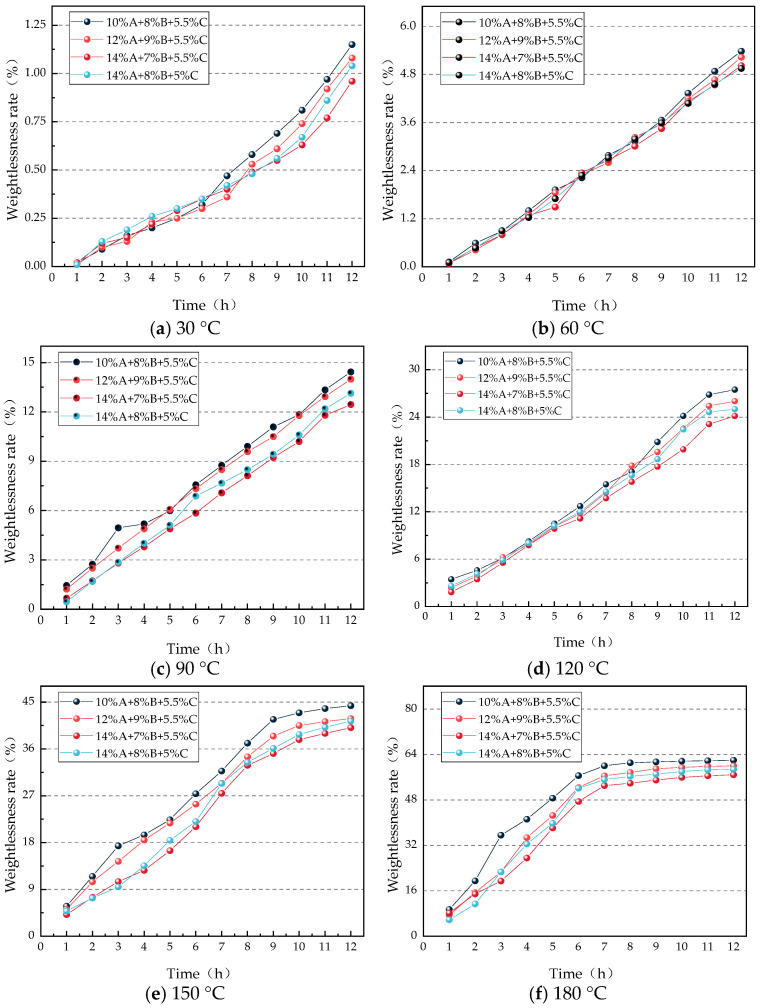
Variation Curve of Gel Weight Loss Rate under Different Constant Temperature Conditions.

Based on the formula, the experimental data were calculated, and the change curve of gel weight loss rate was plotted, as shown in Figure 2.

**Figure 3:**
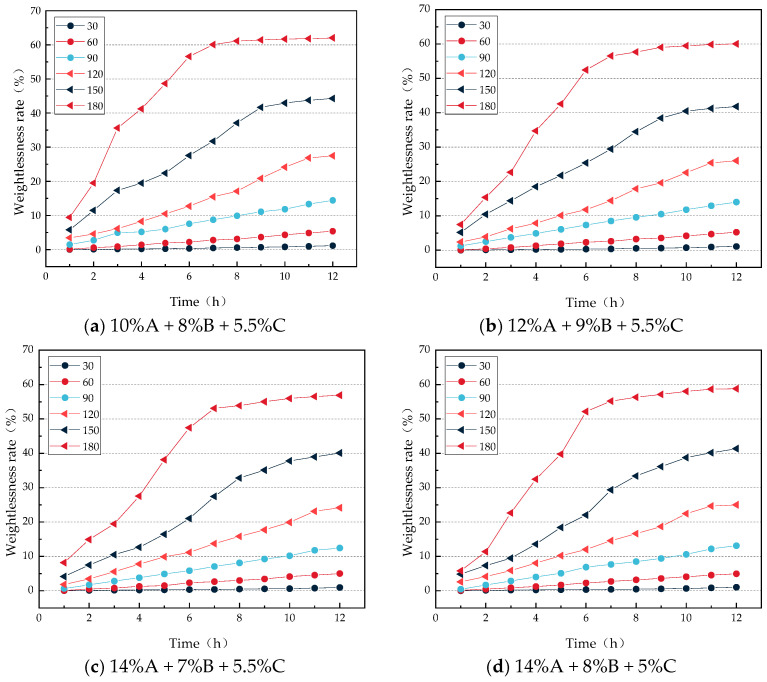
Curve of weight loss rate changes of gels with different ratios under constant temperature conditions.

As shown in Figure 2, the weight loss rate of the gel gradually increases with rising temperature. At 30 °C, 60 °C, and 90 °C, the weight loss rate of the gel steadily rises over the 0–12 h period. At 120 °C, the gel undergoes rapid weight loss within 0–11 h, after which the rate of weight loss slows and stabilizes between 11 and 12 h. At 150 °C, rapid weight loss occurs within 0–9 h, followed by a slowdown and stabilization from 9 to 12 h. At 180 °C, the gel shows rapid weight loss in the first 6 h, and the rate of loss decelerates and stabilizes between 6 and 12 h. This behavior can be attributed to the fact that at lower temperatures, water evaporation is slower, allowing the gel network structure to effectively retain moisture, resulting in a relatively stable weight loss process. Under moderately elevated temperatures, although evaporation accelerates, the structural stability of the gel network initially resists rapid moisture loss, maintaining a steady weight loss rate for a certain period. However, when the temperature exceeds 120 °C, the gel network gradually degrades, leading to a noticeable increase in weight loss until a new equilibrium is reached, at which point the weight loss rate stabilizes. Furthermore, comparing the weight loss rates of the four gel formulations across different temperatures, the formulation with 14% A + 7% B + 5.5% C exhibits the lowest weight loss rate at all temperatures, indicating that this formulation forms a more stable internal network structure and provides the best water retention performance. The weight loss trends of the four gel formulations are compared in Figure 3.

As shown in Figure 3, the weight loss rate of the gel samples gradually increases over time under different temperature conditions. Specifically, at 30 °C, 60 °C, and 90 °C, the four groups of gels exhibit relatively small changes in weight loss rate, with limited water loss, generally remaining below 15%. When the temperature reaches 120 °C, the weight loss rates of the four gel groups begin to increase significantly. After 12 h of testing at 120 °C, the average weight loss rate is 25.67%; at 150 °C, the average weight loss rate reaches 41.88%; and at 180 °C, it rises to 59.43%. This phenomenon may be attributed to structural changes in the gel as temperature increases, which facilitate the escape of moisture. Eventually, however, the gel structure reaches a new equilibrium state, and weight loss stabilizes.

#### 2.2.2. Thermal Stability Test Analysis Under Heating Conditions

**Figure 4:**
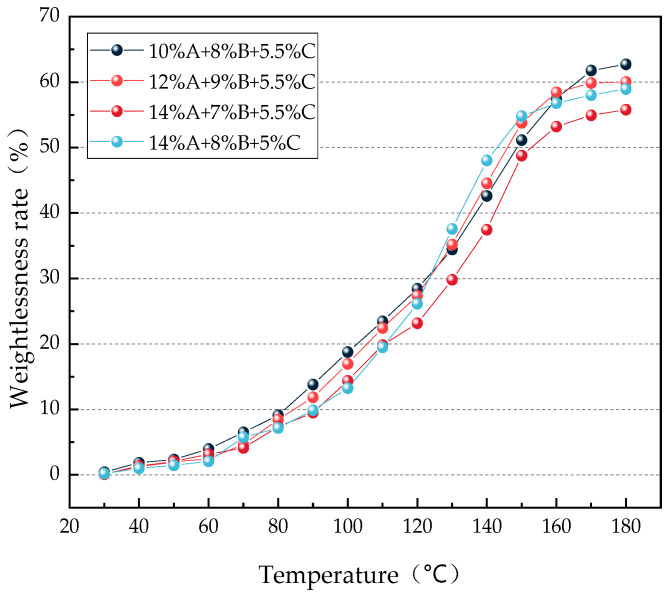
Curve of Gel Weight Loss Rate Changes under Heating Conditions.

The gel weight loss rate curve under heating conditions is shown in Figure 4.

As shown in Figure 4, during the heating process, the four groups of gel samples with different formulations exhibit similar trends in weight loss rate: under conditions of 30 °C to 60 °C, the weight loss rate of the gels changes only slightly; between 60 °C and 140 °C, the gels undergo a rapid weight loss phase; and from 140 °C to 180 °C, the weight loss rate tends to stabilize. This is because, at lower temperatures, the gel effectively retains moisture, slowing down the rate of evaporation. However, as the temperature continues to rise, the evaporation of water from the gel accelerates, and the internal network structure of the gel begins to degrade, leading to a gradual increase in weight loss rate. When the temperature exceeds 140 °C, most of the water has already evaporated, and further weight loss is primarily caused by additional breakdown of the gel network. At this point, the gel reaches a new equilibrium state, resulting in stabilization of the weight loss rate. Additionally, it can be observed from the figure that the gel with the formulation 14% A + 7% B + 5.5% C exhibits an overall lower weight loss rate compared to the other three groups, indicating that the gel with this concentration ratio possesses a more stable network structure and demonstrates the lowest weight loss rate under high-temperature conditions.

Based on the viscosity tests, exudation tests, strength tests, and thermal stability tests of the four gel formulations, it can be seen that the gel with the composition of 14% A, 7% B, and 5.5% C exhibited relatively superior performance. Therefore, the optimal gel formulation was finally determined to be 14% A, 7% B, and 5.5% C.

### 2.3. Characterization of the Microscopic Structure of Gels

**Figure 5:**
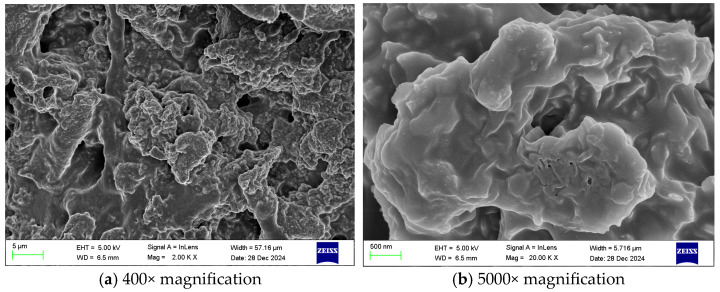
Scanning electron microscope images of the gel at different magnifications.

**Figure 6:**
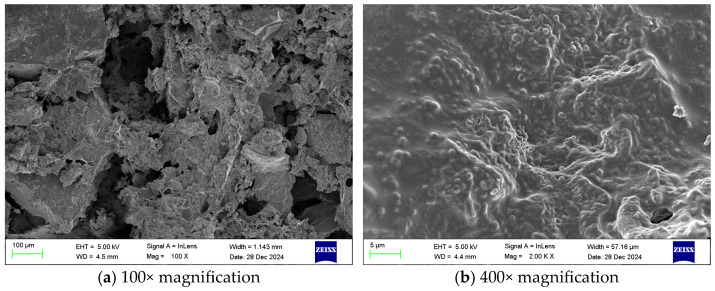
SEM images of gel coal samples at different magnifications.

Figure 5 shows SEM images of the gel at different magnifications. Figure 6 shows SEM images of the coal sample gel at different magnifications.

As shown in Figure 5, the gel material appears overall uniform and continuous, with a smooth surface and no significant cracks or defects, indicating that the gelation process is effective. At the same time, the components are closely connected, forming a dense network structure that enhances the internal stability of the gel and gives it high strength. During the prevention of spontaneous coal combustion, this gel can withstand greater pressure, thereby improving its fire prevention and suppression performance. In addition, there are a large number of pore structures within the gel; these pores are evenly distributed and interconnected, which not only helps improve the gel’s adsorption capacity but also enhances its thermal stability and flame retardant properties [20].

Combining the scanning electron microscope images at different magnifications in Figure 5 and Figure 6, it can be seen that the gel material uniformly covers the surface of the coal samples, effectively encapsulating them and forming a dense adhesive layer, demonstrating the gel’s excellent encapsulation performance. This encapsulation effect not only effectively prevents contact between coal and oxygen, thereby reducing oxidation reactions, but also enhances overall stability and improves flame-retardant properties. In addition, the gel maintains a good gelling effect, with a uniform and continuous morphology; the network structure is dense, and the internal components are tightly interwoven; the pore structure is abundant, evenly distributed, and interconnected.

### 2.4. Carbon Dioxide Adsorption Performance of the Gel

#### 2.4.1. CO_2_ Adsorption Isotherm

(1) Variation of gel CO_2_ adsorption with pressure

**Figure 7:**
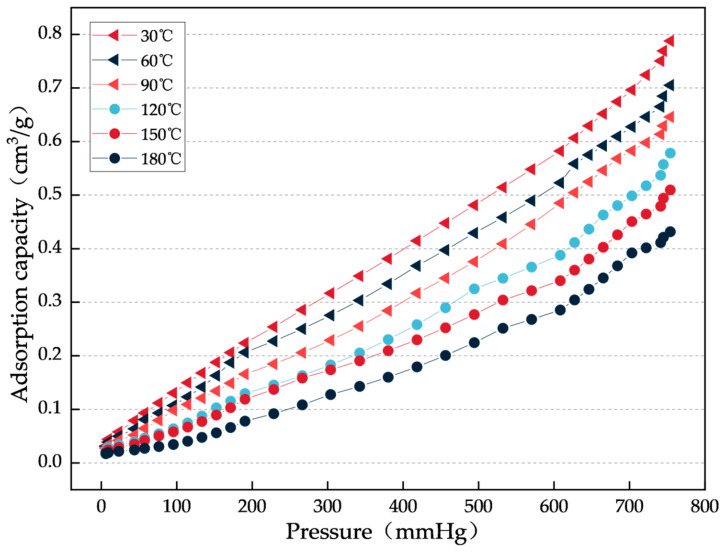
Curve of Gel CO_2_ Adsorption Capacity versus Pressure under Different Constant Temperature Conditions.

To investigate the maximum adsorption capacity of the gel at equilibrium, the relationship between its CO_2_ adsorption amount and pressure was measured at a constant temperature of 30 °C, with the results shown in Figure 7.

As shown in Figure 7, the adsorption isotherm of CO_2_ on the gel exhibits typical Langmuir-type characteristics: in the low-pressure region, the adsorption amount increases slowly with pressure; in the high-pressure region, the adsorption amount increases rapidly and gradually approaches saturation. This indicates that the gel prefers monolayer adsorption of CO_2_. The experimental results clearly demonstrate the decisive effect of pressure on the gel’s CO_2_ adsorption performance.

(2) CO_2_ Adsorption Kinetics

**Figure 8:**
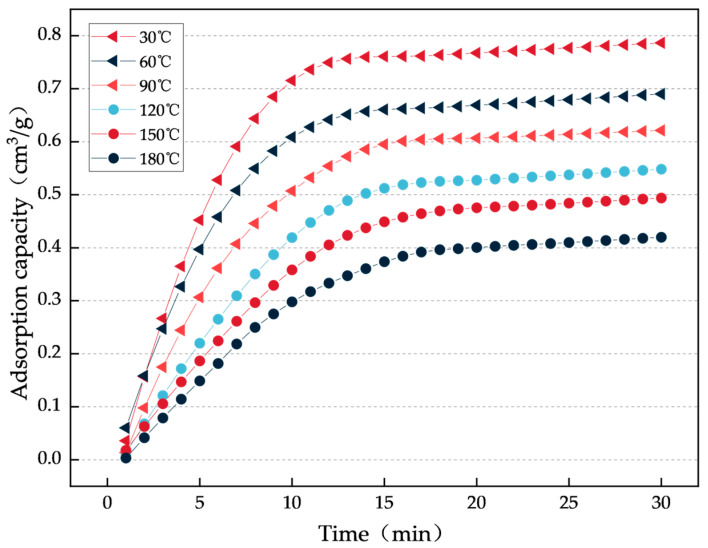
Graphs of gel CO_2_ adsorption over time under different constant temperature conditions.

The adsorption rate is crucial for the practical application of fire-extinguishing materials. To this end, under constant conditions at atmospheric pressure and 30 °C, the change in the gel’s adsorption capacity for CO_2_ over time was studied to evaluate its adsorption kinetics, as shown in Figure 8.

From the data in Figure 8, The adsorption rate is crucial for the practical application of fire-extinguishing materials. To this end, under constant conditions at atmospheric pressure and 30 °C, the change in the gel’s adsorption capacity for CO_2_ over time was studied to evaluate its adsorption kinetics, compared with the CO_2_ adsorption amount of the gel at 30 °C, the adsorption decreases by 12.82% at 60 °C, by 21.79% at 90 °C, by 30.77% at 120 °C, by 37.18% at 150 °C, and by 47.44% at 180 °C. In addition, the higher the temperature, the slower the gel reaches adsorption equilibrium. This phenomenon is also related to the thermal motion of gas molecules; as the temperature rises, the increased thermal motion makes it more difficult for gas molecules to be adsorbed by the gel material, delaying the time needed to reach adsorption equilibrium.

(3) Effect of Temperature on CO_2_ Adsorption Performance

Table 5 lists the CO_2_ equilibrium adsorption amounts measured under different isothermal conditions (30 °C to 180 °C) at atmospheric pressure.

As shown in Table 5, as the temperature increases from 30 °C to 180 °C, the CO_2_ equilibrium adsorption capacity of the gel decreases significantly (from 0.78 cm^3^/g to 0.41 cm^3^/g), and the time required to reach adsorption equilibrium is extended at higher temperatures. This phenomenon conforms to the basic principles of physical adsorption, where higher temperatures intensify the thermal motion of gas molecules, which is unfavorable for adsorption. The results indicate that although high temperatures reduce its adsorption capacity, the gel still maintains a certain level of adsorption ability over a wide temperature range, demonstrating good thermal stability and the capability to adapt to complex downhole thermal environments.

#### 2.4.2. Temperature-Programmed Desorption Test

**Figure 9:**
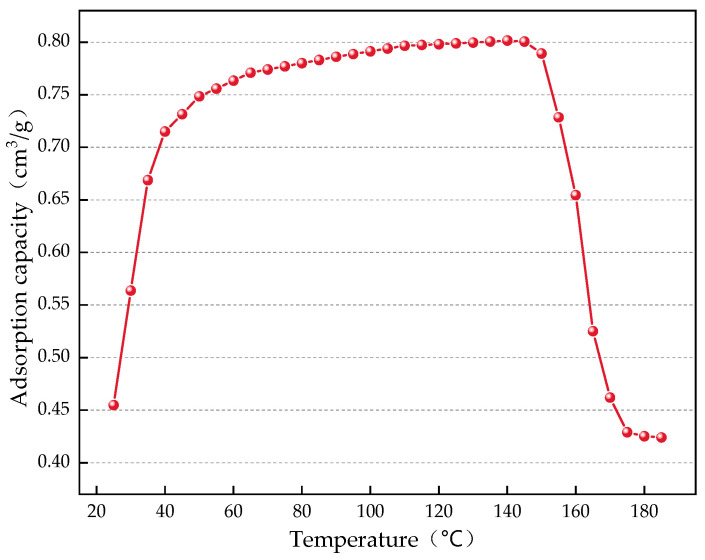
Graph of CO_2_ adsorption changes of gel under heating conditions.

The change in the CO_2_ adsorption capacity of the gel material under heating conditions is shown in Figure 9.

As shown in Figure 9, under normal pressure conditions, the adsorption capacity of the gel material for CO_2_ increases with the gradual rise in temperature, but the rate of increase gradually slows down. This indicates that the thermal motion of gas molecules makes it more difficult for CO_2_ to be adsorbed by the gel material. When the temperature exceeds 145 °C, the gel material’s ability to adsorb CO_2_ begins to decline sharply. This may be because the structure of the gel material changes at high temperatures, causing some of the previously adsorbed CO_2_ to be released. However, the final CO_2_ adsorption amount tends to stabilize, indicating that after undergoing structural changes, the gel material reaches a new equilibrium state, thereby maintaining its capacity to adsorb CO_2_.

#### 2.4.3. Cyclic Adsorption Test Method

**Figure 10:**
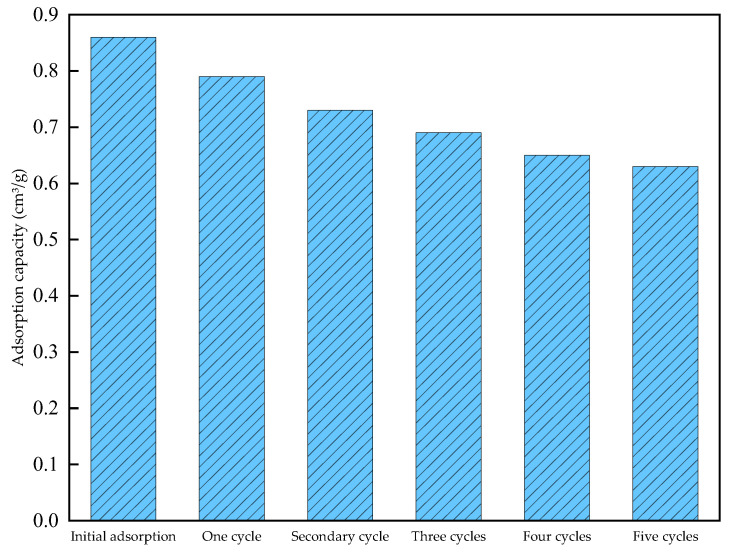
Gel CO_2_ Cyclic Adsorption Capacity Trend Chart.

The CO_2_ adsorption performance of the gel material was tested over 5 cycles, and the results are shown in Figure 10 and Table 6.

Based on the data from Figure 10 and Table 6, it can be seen that under room temperature and normal pressure conditions, the CO_2_ adsorption capacity of the gel material decreases after cycling. This may be due to changes in the chemical stability and physical structure of the gel material during the cycling process, which reduces its ability to capture CO_2_ molecules and thus affects its cycling efficiency. However, overall, after five cycles, the average adsorption capacity of the gel material is 0.73 cm^3^/g, and the average cycling efficiency reaches 81.16%, the recycling efficiency of literature in the same field is 60%, which is an increase of 21.16% compared to it [21]. indicating that the gel material exhibits good stability and reusability during cyclic use.

### 2.5. Analysis of Gel Inhibition on Spontaneous Combustion Characteristics of Coal

#### 2.5.1. Isothermal Adsorption Test

During the process of coal spontaneous combustion, as the temperature of the coal gradually rises, CO is the first gas-phase product to be generated and exhibits the most significant changes in concentration. The variation in its volume fraction can directly reflect the intensity of the coal oxidation reaction and the release of thermal energy [22]. In addition, the change in CO volume fraction is closely related to temperature, and by monitoring the CO volume fraction, the progress of coal spontaneous combustion can be determined more accurately, and the spontaneous combustion temperature can be predicted. Therefore, CO was chosen as the primary indicator gas to record the variation of its volume fraction with temperature, in order to study the effect of gels with different contents on the inhibition of coal spontaneous combustion. The changes in CO volume fraction for raw coal and each gel treatment group are shown in Figure 10.

**Figure 11:**
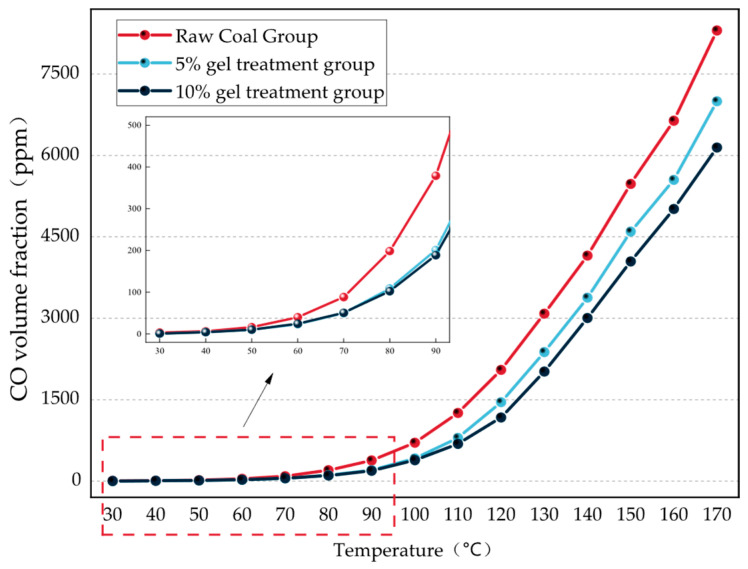
CO Volume Fraction Change Curve.

As shown in Figure 11, the trend of CO volume fraction variation for the raw coal group and the groups treated with different gel contents is generally consistent, both exhibiting an exponential growth pattern. In the raw coal group, the CO volume fraction is relatively low within the temperature range of 30–70 °C, indicating that the reaction rate between coal and oxygen is relatively slow in this temperature range, releasing fewer gaseous products and thus posing a lower risk of spontaneous combustion. When the temperature reaches 70 °C, the CO volume fraction of the samples begins to increase sharply, indicating that as the temperature continues to rise, the samples are in a state of continuous oxidation and heat accumulation, and the coal–oxygen reaction becomes more intense, producing more gaseous products. When the temperature exceeds 120 °C, the coal–oxygen reaction intensifies further, and the CO volume fraction shows a more pronounced exponential increase. Compared with the raw coal group, in the gel-treated groups, the CO volume fraction of the samples only begins to rise sharply after the temperature reaches 80 °C, indicating that the gel partially suppresses the intensity of the coal–oxygen reaction, requiring a higher temperature for the reaction to become more vigorous. Furthermore, Figure 10 also shows that compared with the raw coal group, the CO volume fraction of the gel-treated coal samples is significantly reduced, and the rate of increase with temperature is weaker. Additionally, the greater the gel content, the less CO is produced. Under conditions of 170 °C, the raw coal samples produced 8304.77 ppm of CO, while the samples with 5% and 10% gel produced 6998.92 ppm and 6148.07 ppm of CO, representing decreases of 15.72% and 25.97% compared to the raw coal.

In summary, the addition of the gel can slow down the low-temperature oxidation of coal and has a relatively good inhibitory effect on coal spontaneous combustion. Furthermore, as the gel content increases, the inhibitory effect becomes more pronounced. This is because after the gel forms, it creates a protective layer on the surface of the coal. This layer is dense, which not only effectively wraps the coal but also isolates it from contact with oxygen, thereby slowing down the coal oxidation reaction. In addition, the moisture in the gel is not easily lost at low temperatures and can evaporate and absorb heat at high temperatures, allowing the gel to further delay the coal oxidation process and play a significant role in preventing coal spontaneous combustion.

#### 2.5.2. Analysis of Gel Inhibition on Coal Spontaneous Combustion Intensity

By studying the changes in the volumetric fraction of CO released from two groups of samples during the heating process, a qualitative analysis of the gel’s inhibition of coal spontaneous combustion can be conducted [23,24,25]. However, this method cannot accurately assess the flame-retardant effectiveness of the gel under different temperature conditions. To achieve a quantitative analysis of the gel’s inhibition of coal spontaneous combustion, the intensity of the gel’s inhibition can be measured by calculating the CO inhibition rate, which is calculated using Equation (1).

(1) $$\eta =\frac{V_{raw\;coal}-V_{treated}}{V_{raw\;coal}}\times 100\%$$

In the formula, *η*—CO inhibition rate, %;

*V*_raw coal_—CO gas volume fraction in raw coal, ppm;

*V*_treated_—CO gas volume fraction in coal samples treated by each gel experiment group, ppm.

**Figure 12:**
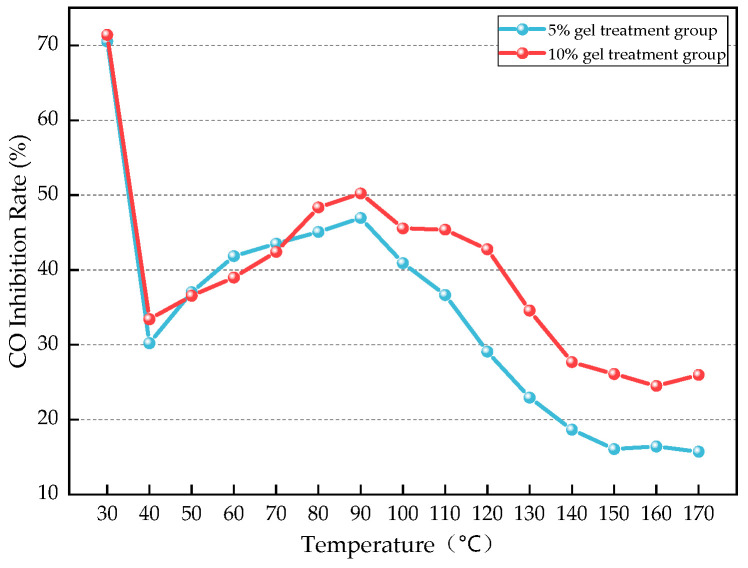
CO Inhibition Rate Change Curve.

**Figure 13:**
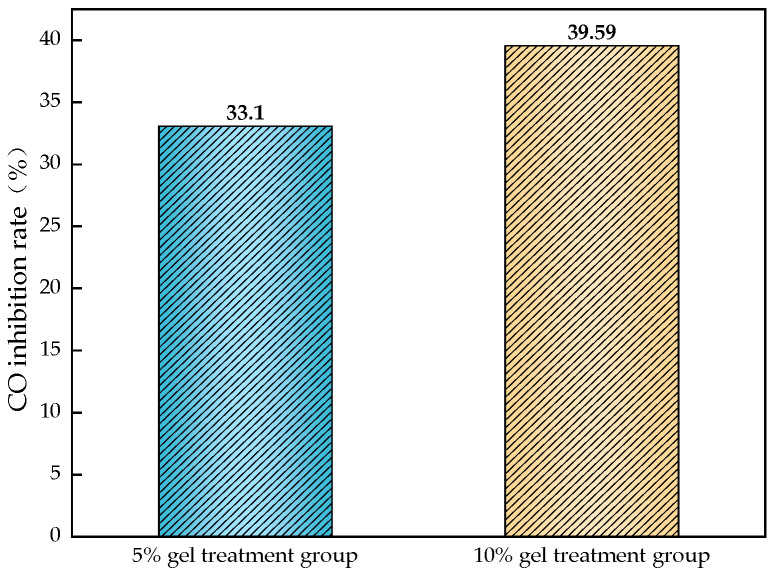
Average CO Inhibition Rate of Each Gel Treatment.

According to Equation (1), the relationship between CO inhibition rate and temperature for the two gel treatment groups was calculated as shown in Figure 12, and the average CO inhibition rate is shown in Figure 13.

As shown in Figure 12, under the condition of 30 °C, the CO inhibition rates of both gels exceeded 70%. This phenomenon is attributed to the fact that the coal oxidation reaction is in a relatively low-activity state at this stage, resulting in a lower CO content and thus a relatively high CO inhibition rate. Within the temperature range of 40–90 °C, the CO inhibition rates of both gels gradually increased with rising temperature, with the CO inhibition rate of the 5% gel reaching a maximum of 46.94% and that of the 10% gel reaching a maximum of 50.22%. This indicates that the addition of the gel effectively reduced the coal oxidation reaction, slowed down its rate, and clearly inhibited spontaneous combustion of the coal. However, when the temperature exceeded 90 °C, the CO inhibition rates of the gels gradually decreased with temperature and eventually stabilized, with the 5% gel stabilizing at around 16% and the 10% gel stabilizing at around 25%. This suggests that under continued high-temperature conditions, the gel has either reached its maximum effect or its internal structure has been damaged, forming a state of equilibrium. Therefore, the inhibitory effect of the gel gradually decreases and eventually stabilizes.

As shown in Figure 13, the gel has a very obvious effect on CO inhibition, and the average CO inhibition effect of the 10% gel is better than that of the 5% gel. This may be because a higher gel content forms a thicker protective layer on the surface of the coal sample, which can more effectively block oxygen and reduce the contact between coal and oxygen, thus resulting in a better inhibitory effect.

#### 2.5.3. Activation Energy Oxidation Kinetics Analysis

Activation energy refers to the minimum energy threshold required for a chemical reaction to occur [26,27]. The lower the activation energy, the easier it is for coal to undergo oxidation at lower temperatures. In addition, the magnitude of activation energy directly affects the rate of chemical reactions; low activation energy means a faster reaction rate. This indicates that under the same conditions, the oxidation reaction rate of coal is faster, leading to rapid heat release and a higher risk of spontaneous combustion. Therefore, by measuring the changes in activation energy during the spontaneous combustion of coal, the tendency and reaction rate of coal to spontaneously combust at different temperatures can also be quantitatively analyzed. Based on the reaction rate formula and the Arrhenius equation, the volume fraction of CO produced in the coal oxidation reaction can be analyzed, yielding the calculation formula for the activation energy of the reaction (2):

(2) $$V_{O_{2}}=\frac{V_{CO}}{m}=AC_{O_{2}}\exp(-\frac{E}{RT_{i}})$$

In the formula, $V_{O_{2}}$—oxygen consumption rate, mol/(cm^3^∙s);

*V*_CO_—CO generation rate, mol/(cm^3^∙s);

m—stoichiometric coefficient of CO;

A—pre-exponential factor;

$C_{O_{2}}$—O_2_ volume fraction, ppm;

*E*—apparent activation energy, J/mol;

*R*—universal gas constant, taken as 8.314 J/(mol∙K);

*T*_i_—absolute temperature, K.

Assuming that during the programmed heating experiment, the temperature distribution inside the sample container remains uniform, the gas flow direction stays unidirectional, and the sample mass remains constant before and after the experiment, the CO generation rate equation can be expressed as Equation (3):

(3) $$SV_{CO}\mathrm{dx}=kV_{g}dC_{CO}$$

In the formula, *S*—cross-sectional area of the sample tank, m^2^; *k*—unit conversion factor, taken as 22.4 × 10^9^; *V*_g_—airflow rate inside the sample tank, m/s; *C*_CO_—CO volume fraction, ppm. By combining Equations (2) and (3) and rearranging, Equation (4) can be obtained:

(4) $$\ln C_{CO}=-\frac{E}{R}\cdot \frac{1}{T_{i}}+\ln(\frac{mSAC_{o_{2}}^{n}}{kV_{g}})$$

According to Equation (4), when the gas flow rate remains constant, $\ln(\frac{mSAC_{o_{2}}^{n}}{kV_{g}})$ can be regarded as a constant. At this time, there is a linear relationship between lnC_CO_ and 1/Ti. By taking lnC_CO_ as the vertical axis and 1/*T*_i_ as the horizontal axis to construct a Cartesian coordinate system, a straight line can be obtained through data fitting. Calculating the slope of this straight line allows for the determination of the apparent activation energy E of the coal sample.

**Figure 14:**
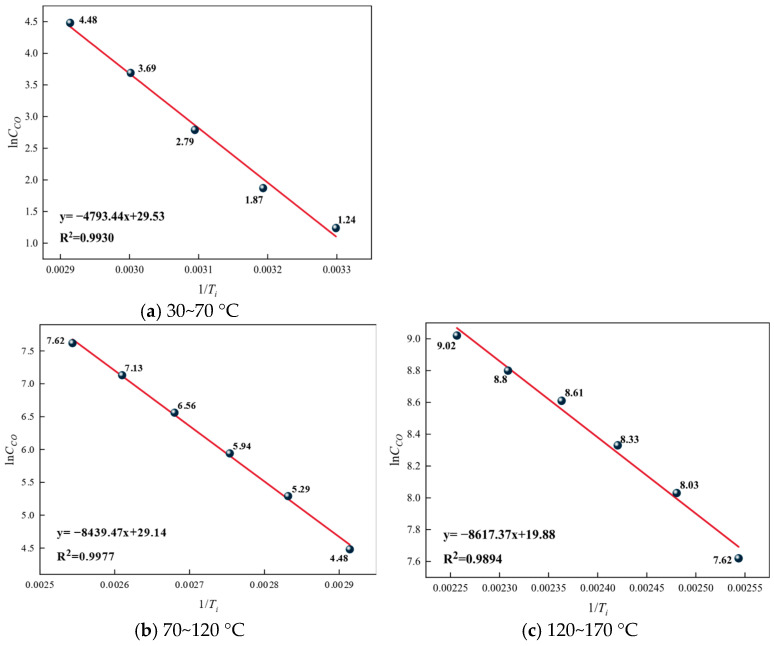
Relationship between Raw Coal lnC_CO_ and 1/*T*_i._

**Figure 15:**
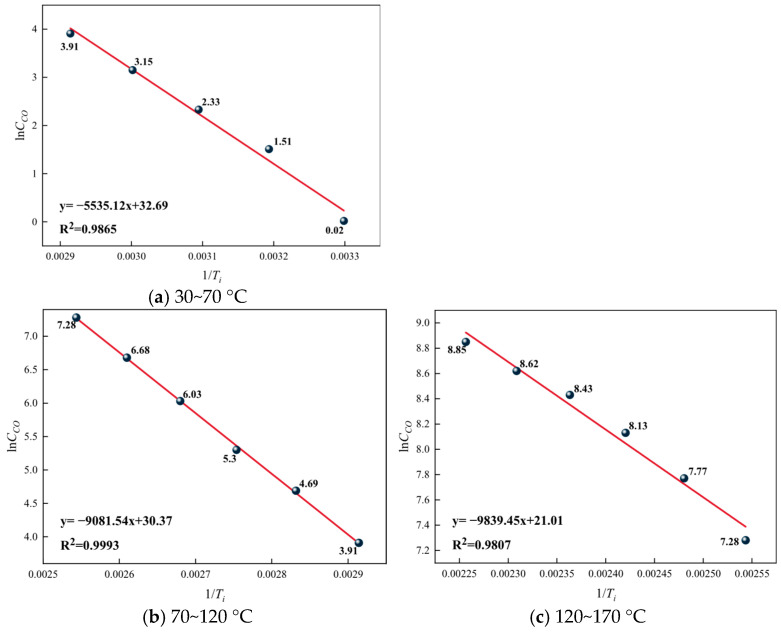
Relationship between lnC_CO_ and 1/*T*_i_ in the 5% gel treatment group.

Based on the analysis of CO concentration and generation rate changes, the critical temperature of coal spontaneous combustion is determined to be 70 °C, and the dry cracking temperature is 120 °C. Considering that the rate of the oxidation reaction changes sharply at these two characteristic temperature points, the temperature range is divided into three stages to determine the apparent activation energy of the reactions for each group of coal samples: the stage before the critical temperature (30–70 °C), the stage from the critical temperature to the dry cracking temperature (70–120 °C), and the stage after the dry cracking temperature (120–170 °C). The lnC_CO_ versus 1/*T*_i_ relationship graphs for different temperature ranges of the raw coal samples are shown in Figure 13, for the 5% gel-treated samples in Figure 14, and for the 10% gel-treated samples in different temperature ranges in Figure 15.

**Figure 16:**
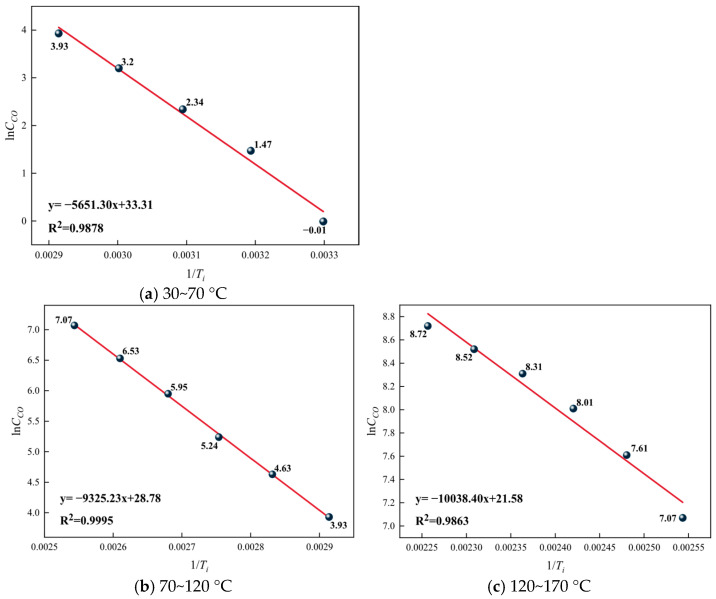
Relationship between lnC_CO_ and 1/*T*_i_ in the 10% gel treatment group.

**Figure 17:**
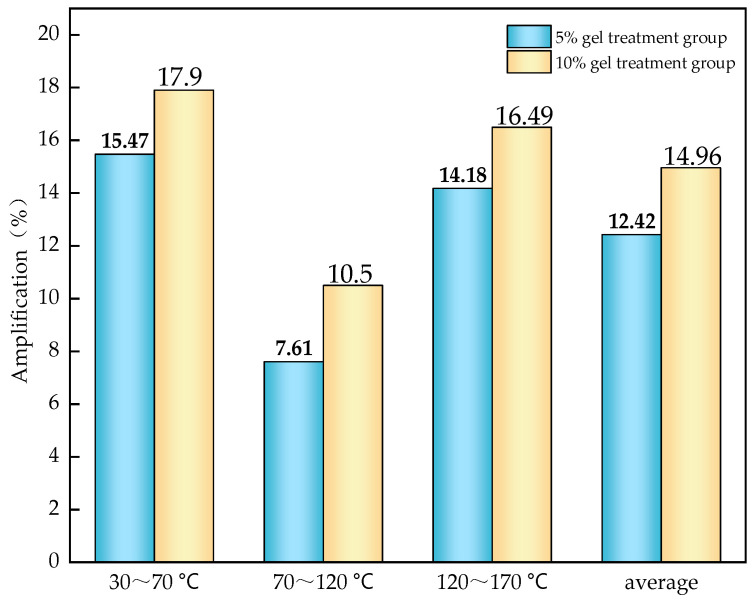
Activation energy increase of each sample group at different temperature ranges.

From Figure 14, Figure 15 and Figure 16, it can be seen that in different temperature ranges, the correlation coefficients R^2^ of the fitted curves for each group of samples are all greater than 0.9, indicating that the fitted lines can effectively explain and represent the activation energy-related data. Based on the slopes of the fitted lines, further calculations can be carried out to obtain the corresponding activation energies for each group. The results are shown, and the increase in activation energy for each gel treatment group is illustrated in Figure 17.

From Table 7, it can be seen that the apparent activation energy of each group of samples gradually increases with the rise in temperature, but this increasing trend slows down progressively. This is because as the temperature rises, the thermal motion within coal molecules intensifies, and chemical bonds begin to break, requiring more energy, which manifests as an increase in activation energy. However, as the temperature continues to rise, the number of reactive groups within the coal molecules decreases, slowing the trend of activation energy increase. From Figure 17, it can be observed that across different temperature ranges, the activation energy of the gel-treated groups is higher to varying degrees compared with the raw coal group. Specifically, the average increase in activation energy for the 5% gel-treated group is 12.42%, while for the 10% gel-treated group it is 14.96%. This indicates that the addition of gel can increase the activation energy required for coal–oxygen reactions, making coal self-ignition more difficult, and the higher the gel content, the better its effect in inhibiting coal self-ignition.

## 3. Conclusions

A multifunctional gel material with both fire prevention and extinguishing properties and CO_2_ adsorption performance was prepared, and the micro-morphology, CO_2_ adsorption performance and coal spontaneous combustion suppression characteristics of the gel were analyzed in detail. The following conclusions were drawn:

(1) We determined the optimal formulation of the CO_2_-adsorbing fire suppression gel. PVA, PEI, and PAC were selected as base materials, and an orthogonal experiment was designed with three factors and three levels (PVA: 10%/12%/14%, PEI: 7%/8%/9%, PAC: 4.5%/5%/5.5%). Gel formation time was measured using a funnel dripping timer method, and it was found that the degree of influence was PAC > PVA > PEI. The optimal ratio was finally selected as 14% A, 7% B, 5.5% C.

(2) The gel exhibits good CO_2_ adsorption performance. The CO_2_ adsorption amount increases with pressure (slow increase at low pressure, rapid increase at high pressure) and decreases with rising temperature, with longer adsorption equilibrium times at higher temperatures. When the temperature exceeds 150 °C, the adsorption capacity drops sharply before stabilizing. After five cycles at room temperature and normal pressure, the gel’s average adsorption capacity was 0.73 cm^3^/g, with an average cycle efficiency of 81.16%, showing good stability and reusability.

(3) Programmed heating experiments showed that the gel can slow down the low-temperature oxidation of coal and suppress spontaneous combustion, with higher content leading to more significant suppression effects. The CO inhibition rate increases with rising temperature, but at high temperatures, the suppression effect gradually decreases and stabilizes due to reaching the maximum action or structural damage. Additionally, the gel can increase the apparent activation energy of coal oxidation, making spontaneous combustion more difficult, further confirming its effect in inhibiting spontaneous combustion of coal.

(4) The CO_2_-adsorbing and fire-extinguishing performances of the gel were preliminarily validated herein. Future work should optimize material ratios and conduct experiments under extreme conditions such as high temperature and humidity, as well as based on actual parameters in goaf areas, to meet practical application requirements.

## 4. Materials and Methods

### 4.1. Experimental Materials and Equipment

Polyvinyl alcohol (PVA) (CS, degree of deacetylation ≥ 95%, Shanghai Macklin Biochemical Technology Co., Ltd., Shanghai, China); polyethyleneimine (PEI) (99%, Shanghai Macklin Biochemical Technology Co., Ltd.); polyaluminum chloride (PAC) (99%, Shanghai Yien Chemical Technology Co., Ltd., Shanghai, China); deionized water. All coal samples were collected from the Xihan Xinglong Coal Mine in Shaanxi Province.

The experimental equipment includes an electronic balance (0.01 g), a laboratory electric stirrer (LC-ES-120), a dial-type rotational viscometer, and an electric heating digital constant temperature water bath (LC-WB-2). The electronic balance, laboratory electric stirrer, and electric heating digital constant temperature water bath were all purchased from Lychen Technology Co., Ltd. (Shanghai, China).

### 4.2. Sample Preparation

**Figure 18:**
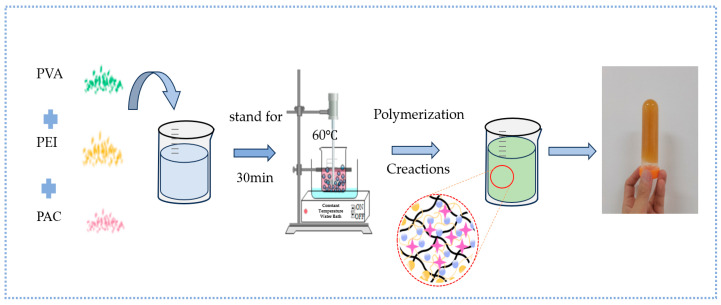
Schematic Diagram of Gel Preparation Process.

In the experiment, a certain mass of polyvinyl alcohol (PVA) and deionized water were first weighed. Subsequently, the weighed deionized water was added into a beaker equipped with a mechanical stirring device. After starting the stirring device, PVA was slowly and uniformly added into the beaker, and the stirring state was continuously maintained to ensure the full dissolution of PVA and avoid precipitation. During the experiment, deionized water was used as the solvent to prepare polyethylenimine (PEI) solutions with mass fractions of 7%, 8% and 9%,, respectively. After PEI was completely mixed with deionized water, the obtained PEI solutions were placed at room temperature for subsequent use. Meanwhile, deionized water was also used as the solvent to prepare polyaluminum chloride (PAC) solutions with mass fractions of 4.5%, 5% and 5.5% respectively. After PAC was completely mixed with deionized water, the obtained PAC solutions were similarly placed at room temperature for subsequent use. After the preparation of the above three solutions, they are evenly mixed in a 1:1:1 ratio. Then, the mixed system was placed in a 60 °C warm water bath to accelerate the polymerization reaction, and finally the new gel was obtained. The schematic diagram of the gel preparation process is shown in Figure 18.

### 4.3. Gel Thermal Stability Test

#### 4.3.1. Thermal Stability Test Under Constant Temperature Conditions

The experiment tests the four groups of gel formulations selected in Section 2.2.2. For each group, 100 g of gel is taken and placed in a thermostatic blast drying oven for continuous heating for 12 h. In the experiment, the temperature of the thermostatic blast drying oven is set at 30 °C, 60 °C, 90 °C, 120 °C, 150 °C, and 180 °C. The samples are weighed every hour, and the weight loss rate of the gels is calculated according to Formula (5).

(5) $$W_{L}=\frac{m_{0}-m_{i}}{m_{0}}\times 100\%$$

where $W_{L}$—water loss rate, %; $m_{0}$—initial mass of the gel, g; $m_{i}$—mass of the gel weighed at a certain time, g.

#### 4.3.2. Thermal Stability Test Under Heating Conditions

The experiment tested the four gel formulations selected in Section 2.2.2. For each gel group, 100 g was taken and placed in a constant-temperature ventilated drying oven. The test started at 30 °C and increased to 180 °C, with the temperature rising by 10 °C every 30 min, and the mass change of the gel at each time point was recorded. The gel’s weight loss rate was calculated according to Formula (5).

### 4.4. Basic Performance Research

#### 4.4.1. Characterization of Gel Microstructure

The sample is processed using vacuum freeze-drying technology, then mounted on the SEM sample stage, ensuring close contact between the sample and the stage. Close the SEM sample chamber door, start the vacuum pump, and evacuate the sample chamber to a high vacuum state to ensure the stability of the electron beam and the cleanliness of the sample. Parameter settings: Acceleration voltage: Choose an appropriate acceleration voltage based on the characteristics of the sample and the required resolution, 15 kV; Working distance: Adjust the distance between the electron beam and the sample surface, usually 5–10 mm; Electron beam current: Select an appropriate electron beam current to ensure sufficient signal strength and image quality. Image acquisition: Choose the appropriate magnification and position for capturing and cropping images, retain the necessary images, record experimental data, and shut down the instrument [13,14,28].

#### 4.4.2. Study on the Carbon Dioxide Adsorption Properties of Gels

(1) Isothermal Adsorption Test

Grind the gel sample with a ratio of 14% A:7% B:5.5% C and pass it through a 100-mesh sieve for later use; place the gel sample under vacuum and set the temperature to 35 °C for 24 h to remove surface-adsorbed gases and moisture.

Then measure the adsorption isotherms: (1) CO_2_ adsorption capacity of the gel with varying pressure: place 0.5 g of gel sample in the adsorption instrument, set the CO_2_ temperature at six different levels: 30 °C, 60 °C, 90 °C, 120 °C, 150 °C, and 180 °C, and gradually increase the CO_2_ pressure to measure the adsorption isotherms, with a pressure range of 0–760 mmHg. (2) CO_2_ adsorption capacity of the gel with varying time: place 0.5 g of gel sample in the adsorption instrument, set the CO_2_ temperature at six different levels: 30 °C, 60 °C, 90 °C, 120 °C, 150 °C, and 180 °C, set the CO_2_ pressure at 760 mmHg, and measure the adsorption isotherms every 1 min, with a test time range of 0–30 min.

(2) Constant Temperature Adsorption Test

Test the vacuum-treated gel material by placing 0.5 g of gel material in the adsorption instrument. Set the test pressure at 760 mmHg, and measure the CO_2_ adsorption of the gel material under rising temperature conditions. The heating rate is 1 °C/min, and CO_2_ adsorption data is recorded every 5 °C increase, with a test temperature range of 25–180 °C.

(3) Cyclic Adsorption Test

Test the vacuum-treated gel material by placing 0.5 g of gel material in the adsorption instrument. Set the test temperature at 20 °C and conduct the adsorption test by gradually increasing the CO_2_ pressure up to a maximum of 760 mmHg to obtain the gel material’s maximum CO_2_ adsorption capacity at ambient temperature and pressure. After the pressure reaches 760 mmHg, simulate desorption conditions by lowering the pressure to release the adsorbed CO_2_ from the gel material. After completing the first desorption, conduct the adsorption test again, repeating this process multiple times to evaluate the gel material’s performance during cyclic operation.

### 4.5. Study on the Inhibition of Coal Spontaneous Combustion by Gel

**Figure 19:**
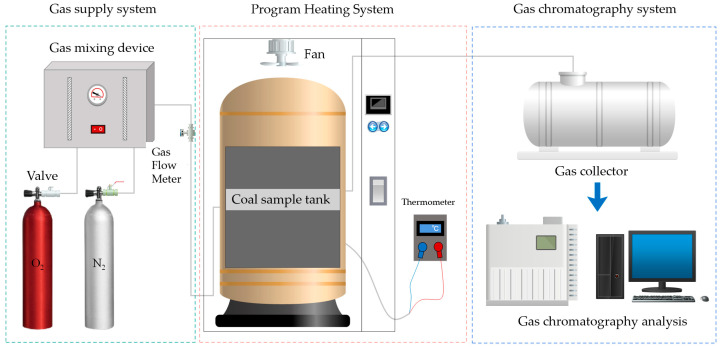
Schematic Diagram of Programmed Heating Flame Retardant Performance Test.

The programmed temperature rise experiment gradually increases the environmental temperature to simulate the heating conditions and environment during coal’s low-temperature oxidation and spontaneous combustion process. It measures the concentrations of gas products such as CO and CH_4_ released by coal under different temperature conditions, thereby analyzing characteristic parameters of the coal samples such as critical temperature and oxidation temperature [29,30]. Conducting a programmed temperature rise experiment on both gels and coal samples can evaluate the flame-retardant effect of gel materials on coal under different temperature conditions and verify the flame-retardant performance of the gel. A schematic diagram of the programmed temperature rise flame-retardant performance test is shown in Figure 19.

(1) Sample Preparation During the programmed heating experiments, all coal samples were collected from the Xinglong Coal Mine in Xihan, Shaanxi Province. To ensure the accuracy and standardization of the experimental results, the analysis strictly follows the relevant provisions of the ‘Methods for Sampling Coal from Coal Seams’ ensuring that the size of the coal block taken is not less than 100 mm × 100 mm × 100 mm. After sampling, the coal samples should be immediately sealed and packaged to prevent oxidation and deterioration. After sampling, the coal samples were immediately sealed to prevent oxidation and deterioration. Labels were attached to the outside of the packaging, and then the samples were placed in ordinary packaging bags for secondary sealing to preserve their original state.

The coal samples were crushed using a jaw crusher and then sieved by particle size, selecting five different particle size ranges: 0–0.9 mm, 0.9–3 mm, 3–5 mm, 5–7 mm, and 7–10 mm. Each size fraction of coal was carefully sorted and stored for subsequent experiments.

For the programmed heating experiments, 200 g of coal from each of the five particle size ranges was mixed. The raw coal served as the control group, while coal samples treated with 5% and 10% gel material served as the experimental groups, making a total of three groups. In the gel-treated groups, after mixing, the coal samples were allowed to stand at room temperature for 24 h to allow sufficient reaction between the gel and the coal. Then, the five groups of coal samples were dried in a drying oven at 35 °C for 12 h before testing.

(2) Sample Loading and Leak Testing

The dried samples were placed in a coal sample container, connected to the instrument and pipelines, and the air pump was started to continuously introduce airflow. Gas tightness was then tested. Once a good seal was confirmed, the air flow was adjusted to 120 mL/min and maintained for 20 min to remove any residual air in the apparatus.

(3) Data Collection

The test temperature range was 30–170 °C with a heating rate of 1 °C/min. Gas sampling was conducted every time the temperature increased by 10 °C. During each sampling, five tubes of gas were collected: the first three tubes were naturally vented, the fourth tube was used for subsequent testing, and the fifth tube was kept as a backup. The collected gas samples were injected into a gas chromatograph to determine their composition and content, and the relevant data were recorded.

(4) Cooling

After the experiment, the programmed heating oven was allowed to cool naturally, and airflow continued for a while before eventually turning off the air pump.

## Figures and Tables

**Table 1:** Factor Level Design Table.

Level	Factor		
	A	B	C
	PVA Content (%)	PEI Content (%)	PAC Content (%)
1	10.0	7.0	4.5
2	12.0	8.0	5.0
3	14.0	9.0	5.5

**Table 2:** Orthogonal Experimental Design.

Experiment Number	PVA Content (%)	PEI Content (%)	PAC Content (%)
1	10.0	7.0	4.5
2	10.0	8.0	5.5
3	10.0	9.0	5.0
4	12.0	7.0	5.0
5	12.0	8.0	4.5
6	12.0	9.0	5.5
7	14.0	7.0	5.5
8	14.0	8.0	5.0
9	14.0	9.0	4.5

**Table 3:** Orthogonal Experiment Results.

Experiment Number	PVA Content (%)	PEI Content (%)	PAC Content (%)	Gelation Time (min)
1	10.0	7.0	4.5	20.40
2	10.0	8.0	5.5	9.95
3	10.0	9.0	5.0	15.55
4	12.0	7.0	5.0	13.45
5	12.0	8.0	4.5	16.15
6	12.0	9.0	5.5	9.00
7	14.0	7.0	5.5	8.20
8	14.0	8.0	5.0	9.35
9	14.0	9.0	4.5	13.25

**Table 4:** Significance Analysis of the Gelation Time with Respect to PVA, PEI, and PAC Content.

Source of Difference	Sum of Squared Deviations	Degree of Freedom	Mean Square	F	*p*-Value
PVA	40.056	2	20.028	23.258	0.041
PEI	7.389	2	3.694	4.290	0.189
PAC	84.389	2	42.194	49.000	0.020

R^2^ = 0.987, *p* < 0.05, significant.

**Table 5:** Effect of Adsorption Temperature on CO_2_ Adsorption Capacity of Gel.

Temperature (°C)	CO_2_ Adsorption Capacity (cm^3^/g)
30	0.78
60	0.68
90	0.61
120	0.54
150	0.49
180	0.41

**Table 6:** Cyclic adsorption capacity of the gel at 25 °C.

Number of Cycles	Adsorption Capacity (cm^3^/g)	Cycle Efficiency
0	0.86	/
1	0.79	91.86%
2	0.73	83.88%
3	0.69	81.23%
4	0.65	77.58%
5	0.63	76.25%
Mean	0.73	81.16%

**Table 7:** Activation energy of samples in each group at different temperature ranges.

Temperature Range (°C)	Activation Energy (KJ/mol)		
	Raw Coal Group	5% Gel Treatment Group	10% Gel Treatment Group
30~70	39.85	46.02	46.98
70~120	70.17	75.50	77.53
120~170	71.64	81.81	83.46

## Data Availability

Date are contained within the article.
